# Postoperative Pain, Analgesic Choices, and Ileus: A Snapshot from a Teaching Hospital in a Developing Country

**DOI:** 10.1055/s-0042-1755623

**Published:** 2022-09-02

**Authors:** Ameer Al-Jasim, Alaa A. Aldujaili, Ghaith Al-Abbasi, Hasan Al-Abbasi, Saif Al-Sahee

**Affiliations:** 1 Department of Surgery, Baghdad Teaching Hospital, Medical City Complex, Baghdad, Iraq; 2 Department of Anesthesiology, Al-Alwaiya Maternity Teaching Hospital, Baghdad, Iraq; 3 Department of Surgery, Al-Yarmuk Teaching Hospital, Baghdad, Iraq; 4 Department of Medicine, Baghdad Teaching Hospital, Baghdad, Iraq; 5 Department of Surgery, Tunbridge Wells NHS Trust, Tunbridge Wells, United Kingdom

**Keywords:** postoperative, analgesia, pain relief, opioids, postoperative ileus

## Abstract

**Background** Pain relief can be achieved by diversity of methods with analgesics being the basic form of treatment. Analgesic safety and clinical effectiveness are the core factors in determining the analgesic of choice. One adverse effect of concern with opioids is the postoperative ileus (POI).

**Objective** In this study, we looked at the severity of postoperative pain, the type of analgesics used to control the pain, and the incidence of POI at Baghdad Teaching Hospital. We hypothesized that we would find an association between the type of analgesia used and POI.

**Methods** This observational study was conducted among 100 patients who were residents at the general surgery wards of Baghdad Teaching Hospital. A structured questionnaire was employed focusing on types of analgesics, degree of pain control, and the presence of ileus.

**Results** Sixty-nine percent of patients received a combination of opioids and nonopioids. Moderate-to-severe pain was the most commonly reported category on pain scales. More than half of the patients (57%) were found to have POI during their hospital stay and there was a statistically significant association between the type of analgesia and POI development ( *p* =0.001).

**Conclusions** A mix of analgesics (opioids and nonopioids) was the most common regimen at our center. The majority of the surgical inpatients reported having moderate-to-severe pain on both pain scales used in this study. Ileus incidence following abdominal surgeries (61%) was significantly higher than the reported incidence worldwide (10–30%). Postoperative ileus has multifactorial causes, one of which is the use of opioids for pain control. Considering the high incidence of ileus in our center and the association we found between the use of opioids and ileus, further studies should look at the doses of opioids used and whether alternative analgesic methods might result in less ileus.

## Background

Pain relief can be achieved by a diversity of methods with analgesics being the basic form of treatment. The clinical use of different types of analgesics including opioids and nonsteroidal anti-inflammatory drugs (NSAIDs) is the standard through a proposed ladder for different types of pain; for example, the World Health Organization analgesic ladder for cancer pain or its modified one for acute pain in emergency departments and in postoperative situations. 1

The combination of analgesic drugs has been augmented considerably in the last few years. The main concept is to use two or more drugs with different mechanisms of action to achieve synergistic interaction leading to a sufficient analgesic effect at lower doses, therefore reducing the intensity and frequency of adverse events. One example is the combination of NSAIDS and opioids which is more effective at controlling pain with lower doses of opioids. 1 2

In the assessment of clinical effectiveness of analgesics, relief of pain is one of the most important variables. A set of factors must be met in pain relief interventions for optimum outcome. For example, appropriate clinician–patient interactions on the ward encourage patients to communicate unrelieved pain without feeling needy and weak. Quality assurance and frequent monitoring and displaying patient pain ratings are two other factors that improve the outcome. 2 3 4 5

Analgesic safety is yet another important factor, alongside effectiveness, in making the analgesic of choice for postoperative pain relief. The consequences of overtreatment are often overlooked but can be life-threatening. An observation study done by Taylor and coworkers found that there are high rates of oversedation in the first 12 postoperative hours reaching dangerous levels in 72.7% of patients on patient-controlled analgesia. 6

Acute kidney injury (AKI), ileus, and even death are also among the serious complications of inappropriate postoperative use of different classes of analgesics. NSAIDs were found to cause AKI through impairing renal homeostasis in around one in six patients following major abdominal surgeries and is associated in increased postoperative death at 30 days. 7

The clinical and economic consequences of opioids are of concern as well through their adverse effect as postoperative ileus (POI). In fact, recent international data from developed countries have reported the overall consequence of POI to be an added cost of $8,300 and an increase in hospital length of stay (LOS) of 5 days. 8 9 Still, opioid dosages are not thoroughly evaluated and implemented in the settings of most laparoscopic and open operations at our center which may lead to unnecessarily prolonged patient stays.

At our freestanding, urban, university-affiliated center, the overall image of postoperative pain control is hazy and the management of such cases is done on a case-by-case basis without stepping up the official analgesic ladder for acute/postoperative pain.

Knowledge about the effectiveness of pain treatments is crucial to provide quality care for patients admitted to our wards, and to reduce the medical and economic adverse effects of inappropriate use of analgesics.

The purpose of this study is to take first steps in looking at the types of analgesics used at our center and their adverse effects starting with ileus. We hope to collect the evidence for a more rational use of analgesic medications for the future. We hypothesized that there is an association with the type of analgesics used (especially opioids) and the incidence of POI.

## Methods

### Study Design

This observational study was conducted among 100 patients and residents at the general surgery wards of Baghdad Teaching Hospital. For patients to be included in the study, they had to be in their second day (or beyond) postoperatively, and have undergone open general surgery. Patients who have undergone minimally invasive surgery, those with an inpatient stay earlier than 2 days postoperatively and those who have taken opioids 1 month before surgery for any reason were all excluded from the study.

Patients were sampled via a stratified random sampling for the period between October 2019 to January 2020. Gender (male or female) and the type of analgesia being given (opioids, nonopioids, or both) were the characteristics used to stratify the source population into smaller groups. Thus, six strata were eventually reached which were, then, randomly sampled through random number method via using Stat Trek random number generator (duplicates were not allowed) to achieve equal chances for all eligible patients of being selected to participate in the study. The overall response rate was 99.3%.

### Study Questionnaire

A structured questionnaire was employed for data collection purposes. This questionnaire was built in the English language and based on previous similar studies. 9 10 Then a pilot study was implemented at the same center with a sample size of 12 participants. The results of the pilot study were consistent and the reliability statistic on Cronbach's α was 0.859.

The questionnaire contained three main items. First, the type of analgesia given to the patient. This was taken from the information recorded in the patients' files. Patients were, then, categorized into those who were receiving opioids, nonopioids (including acetaminophen, NSAIDs, N-methyl-D-aspartate receptor antagonists, anticonvulsants [e.g., gamma-aminobutyric acid analogues], β-blockers, α-2 agonists, transient receptor potential vanilloid receptor agonists [capsaicin], and glucocorticoids), and both opioids and nonopioids.

Second, the pain estimation scales including the visual analogue scale (VAS) and the numeric pain rating scale (NPRS), both of which were acquired through patients picking the face and number printed on the questionnaire form for the respective scale upon data collection. The numeric values of pain intensities were divided into three groups (mild, moderate, and severe pain) for the NPRS and five groups (none, mild, moderate, severe, very severe, and excruciating pain) for the VAS. The two scales were presented in a sequential manner to all participants with the NPRS being presented first followed by the VAS to eliminate the carryover effect of one scale to another.

Third, the presence of POI which was recorded based on the history of whether or not the patient passed feces/flatus postoperatively along with the presence or absence of bowel sounds which their lack for 72hours or more postoperatively was employed to define ileus.

The questionnaire also included other data such as demographics, cause of surgery, sequence of postoperative day, history of previous surgeries, presence of nausea, vital signs, and wound examination findings making the questionnaire dependent on both, patients (history taking), and researchers (examination findings) for accurate data collection and addressing potential sources of bias, for example, recall bias.

### Data Collection

Data were collected through the aforementioned dates via a face-to-face interview between patients and researchers during the morning when patients were rested and not in distress for accurate pain estimation using the premade questionnaire. Questions with regard to the patient's history were translated into lay language for the patients to understand, then their responses were translated back into English on the questionnaire form with fixed operational definitions of the patients' spoken terms. Items that can only be addressed by the researcher were collected by a thorough examination of patients.

### Statistical Analysis

The collected data were analyzed using Statistical Package for Social Sciences (SPSS) (version 25.0). Descriptive statistics were presented in frequencies and percentages using appropriate tables and figures for the purpose of comparison. For statistical analysis, exact tests were performed to test the significance of between the study variables. Spearman's correlation was used to clarify the type and strength of relationship between ordinal variables. A *p* -value of <0.05 was considered as statistically significant throughout the analysis.

## Results

Of the 100 participants included in the study, the age was ranging from 13 to 83 with a mean (±standard deviation) of 42 (±18). Half of the participants were males and the other half were females. Most of the participants were in their third postoperative day (23%) among a range of postoperative days 2 to 17 with a mean of 5 (±3).

Midline laparotomy was the most common incision among patients (39%), while the collar incision was the second most common type of incisions (12%). An overview of the sociodemographic data are given in Table 1 .

**Table 1:** An overview of the sociodemographic data

Variable	Frequency (percentage)
Age group (y)	
10–29	16 (16)
30–49	37 (37)
50–69	35 (35)
70–89	12 (12)
Gender	
Male	49 (49)
Female	51 (51)
History of previous surgery	
Yes	74 (74)
No	26 (26)
Surgery type	
Abdominal	71 (71)
Nonabdominal	29 (29)

The most common types of analgesics used were both opioids and nonopioids (69%), followed by nonopioids only (26%).

Most patients were assessed as having moderate pain (75%) on the numeric pain scale (NPRS) and severe pain (38%) on the VAS.

**Fig. 1:**
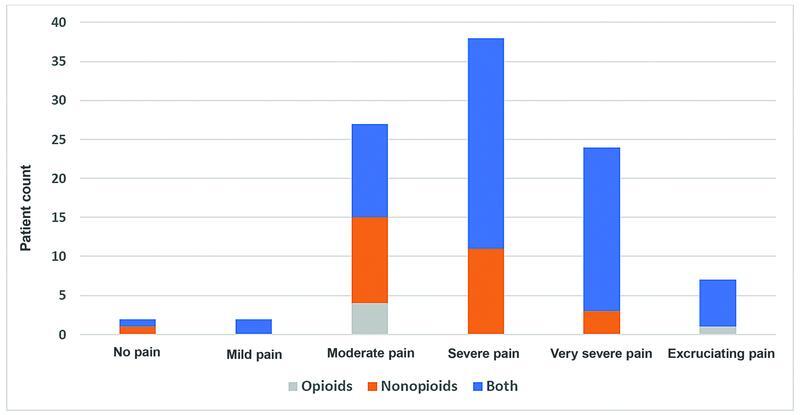
Shows the correlation between the type of analgesia and pain on the visual analogue scale (VAS).

**Fig. 2:**
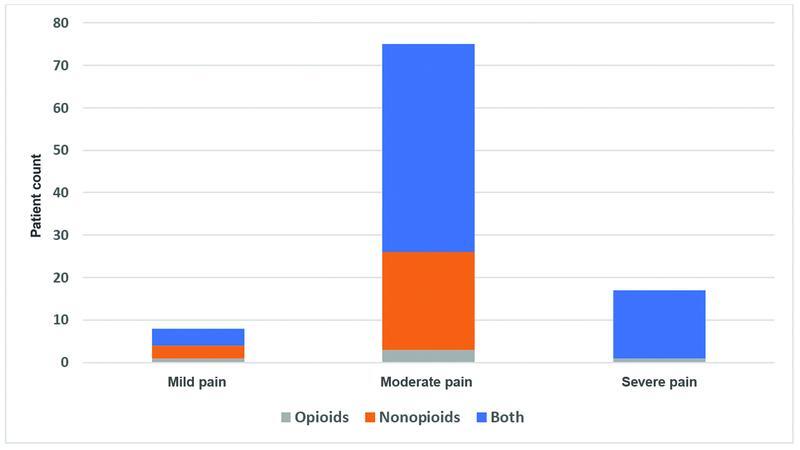
Shows the correlation between the type of analgesia and pain on the numeric pain rating scale (NPRS).

Figs. 1 and 2 show the correlation between the type of analgesia and pain on both VAS and NPRS respectively.

Regarding the effects of the use of different types of analgesia on patients' postoperative mobilization, Table 2 is a crosstabulation showing patients' ability to ambulate at the time of data collection corresponding with their analgesic group.

**Table 2:** Percentages of patients' ability to ambulate across their analgesic groups

Analgesic group	Ambulation	
	Yes	No
Opioids	5 (100%)	0 (0%)
Nonopioids	24 (92.3%)	2 (7.7%)
Mix (opioids and nonopioids)	49 (71%)	20 (29%)

**Fig. 3:**
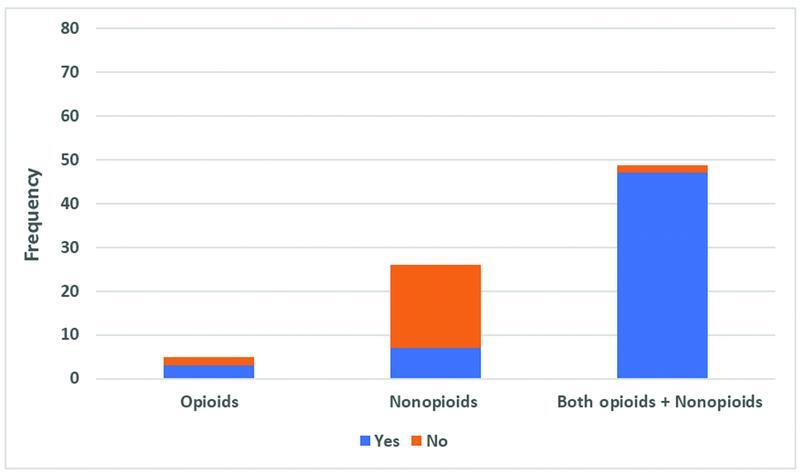
Frequency of participant's ileus development based on their analgesia type given.

More than half of the participants (57%) were found to have ileus during their hospital stay. Sixty percent of those who were receiving opioids developed ileus, while only 27% of those who were given nonopioids developed it. Ileus was identified in 68% of patients who were receiving both types of analgesics ( Fig. 3 ).

**Fig. 4:**
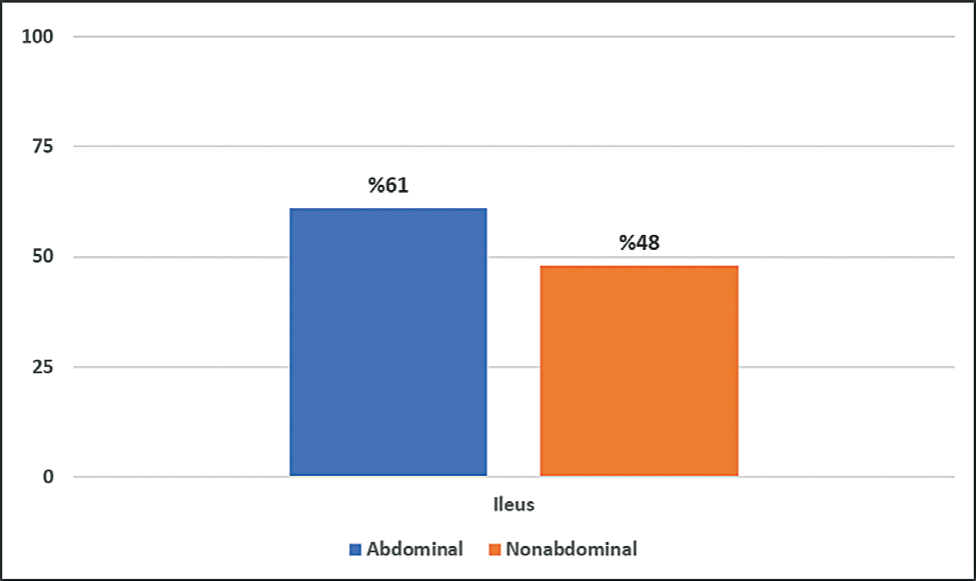
Incidence of ileus in abdominal versus nonabdominal surgeries.

The incidence of ileus according to the type of surgery (abdominal vs. nonabdominal) is shown in Fig. 4 .

There was a statistically significant difference between the type of analgesia used for pain relief and whether patients developed ileus during their hospital stay ( *p* =0.001; Fig. 3 ).

There was a statistically significant difference between ileus development and the degree of pain control on the NPRS ( *p* =0.017) and VAS ( *p* =0.009). The correlation was weakly negative with correlation coefficient (–0.235) on the NPRS and (–0.318) on the VAS.

Ileus development was independent of gender ( *p* =0.545), past surgical history ( *p* =0.602), ambulation postoperatively ( *p* =0.627), and the type of surgery (abdominal vs. nonabdominal; *p* =0.26).

## Discussion

This article discusses general topics related to the analgesia in the postoperative period at Baghdad Teaching Hospital. Most of the participants were in their third postoperative day (23%) among a range of postoperative days 2 to 17. Patients with earlier than 3 days of hospital stay were excluded, as they lack the proper recovery, also for proper assessment of analgesic complications such as ileus. This is in line with the study by Farhadi Hassankiadeh et al which suggested that most of the patients in the general surgery departments have no complications reported leaving the hospital before the third day postoperatively. 11

Midline laparotomy was the most common incision among patients (39%), while the collar incision was the second most common type of incisions (12%). Clay et al in their randomized controlled trial found that patients following a midline laparotomy can have pain reaching up to 7 on the VAS, this pain is more likely to be ameliorated by either scaling up the analgesic choice or using an elastic griddle, if present. 12 Saimanen et al in their search for biological evidence for pain found that levels of the oxidative stress biomarker catalase following midline laparotomy peak immediately in the postoperative period with significant statistical association with the pain on the NPRS. 13

There are many available analgesic agents postoperatively. In this study, they were classified into opioids and nonopioids classes. The majority of the patients (69%) were receiving both opioids and nonopioids to control the pain. Despite opioids are more potent, the addition of nonopioids will still provide 20 to 50% of opioids effect in pain control without opioids-related unwanted effects which delay patients' recovery. 14 15 This fact is still in a debate as Remy et al found that adding nonopioid analgesics will not help in limiting the opioids' side effects because of the requirement of lower doses of individual drugs, yet there was no contradiction regarding the sparing effect of this type of analgesics. 16

The supplemented use of opioids by nonopioids is the best strategy as suggested by previous studies. 15 17 However, in certain situations, the use of potent analgesics is essential to treat acute-moderate-to-severe pain which was the case in 5% of patients in this study. Kehlet and Holte found that opioids-only analgesia is very good in subsiding acute pain but they do not provide dynamic pain control and has significant morbidities after some but not all major procedures. 14

When it comes to exclusive nonopioids analgesia, only 26% of participants were receiving them. It was not a single-type medication but rather a mix of nonopioids. The combination of intravenous paracetamol with a NSAID results in better and longer lasting analgesia in some but not all patients with mild-to-moderate pain postoperatively. 18 Our observations found that opioids are being given to the patients without stepping up a proper analgesic ladder, this can be attributed to many facts including granting the authority of stronger analgesics prescription for on-call junior doctors during night shifts.

Looking at Table 2 , 71% of patients who were given both types of analgesia were able to ambulate indicating good pain control. Regarding those with nonopioids analgesics, only 7.7% were unable to ambulate while all those who were given opioids were able to move freely. These descriptive findings may indicate that pain control is essential for postoperative ambulation. Unfortunately, the extent of pain control required to enhance mobilization cannot be known from the data in hand. Rivas et al examined the hypothesis that postoperative pain is inversely correlated with mobilization and found that lower pain scores are associated with enhanced mobility. Furthermore, their results suggested that improving postoperative analgesia by approximately 3 points on an 11-point Likert's scale might increase mobilization time by as much as 25%, regardless of the type of analgesia used. 19

The extent to which opioid consumption influences postoperative mobilization is still unknown. 19 20 21 22 This study focused on postoperative mobilization in relation with different types of analgesics used and found that the most mobilization percentage is achieved in those who were receiving opioids for pain management. Looking at these percentages for the sake of comparison between analgesic groups is not statistically accurate for the following reasons. First, the surgical indications were different. For example, some patients, who were receiving both types of analgesics, were admitted for an amputation. Second, the inadequate count of patients who were receiving opioids in this study emphasizes the percentage opposite to those who were receiving only nonopioids or both types of analgesics ( Table 2 ). Finally, the difference in the types of nonopioids and their potency for pain control is yet another confounding variable that needs to be controlled for a fair comparison. Thus, concluding that opioids are the best for enhancing postoperative mobilization for patients at our center, and solving the dilemma of opioid consumption that influences on postoperative mobilization is not possible from this study, as these percentages indicate mere values without accounting for the confounding variables and were only demonstrated at descriptive levels. Thus, a well-designed comparative study between the analgesic groups is recommended for a more accurate outcome at our center or others in developing countries.

One of the common postoperative complications is ileus. At our center, the majority of the patients who received opioids analgesics developed ileus (60%), while only 27% of those who received nonopioids developed it ( Fig. 3 ). This is supported by the fact that there was a statistically significant difference among patients in the three groups of analgesics in terms of ileus development. Ferraz et al suggested that NSAIDs not only prevent ileus development compared with opioids but also fasten the resolution and clinical recovery from a preexisted POI. 23 The available literature indicates that the use of opioids is strongly associated with impaired gastrointestinal motility. 24 Therefore, ileus prevention entails minimizing the use of opioids or using other drugs, for example, NSAIDs to limit opioids' side effects through decreasing the dose or even the need to use opioids. 16 The reason that 68% of patients, at our center, who received both opioids and nonopioids analgesics developed ileus is customary for most patients to receive paracetamol vial during the immediate postoperative period of major surgeries for the sake of temperature rather than pain control which is handled by the remaining effect of anesthetics. Thus, patients will still need regular doses of opioids when the half-life of anesthetics has ended to relieve pain hence side effects appear.

The statistically significant association between ileus development along with the degree of pain control on both scales signifies that the pain control done by giving different types of analgesia is complicated by ileus development. This correlation has been shown to be a negative one. Although being weak, it means that the greater the ileus, the less pain reported on the scales and this indicates that analgesics and, especially, opioids are being given for the sole reason of pain relief without accounting for ileus, thus the high incidence of ileus at our center. Frants et al in their randomized clinical trial have highlighted the fact that NSAIDs are an acceptable and safe alternative to opioids for postoperative analgesia in rhinoplasty patients as an example. 3

It is worth noticing that ileus development was independent of gender ( *p* =0.545) and past surgical history ( *p* =0.602), the latter was positive in 74% of the patients participating in this study. Artinyan et al found them to be independent as well with *p* =0.81 and 0.43, consecutively. 25

As shown in Fig. 4 , ileus incidence among those who underwent an abdominal surgery during the current presentation is higher than those who had a surgery elsewhere (nonabdominal). The reported rate of ileus incidence varies among different authors and specialties but is generally between 10 and 30% for abdominal surgery. 26 27 28 29 30 31 This indicates that the reported rate of ileus development at our center is significantly higher than the rate reported by authors in other centers worldwide with regard to abdominal surgeries. This finding might be due to the concept that surgical inpatients at our center are being overdosed with opioids leading to an increased incidence of POI relative to other centers.

The statistical association between ileus development and the type of surgery yielded a nonstatistically significant *p* -value indicating ileus development at our center is independent from the type of surgery (whether abdominal or nonabdominal). This finding supports the aforementioned concept that POI at our center is being primarily influenced by the opioids' effects rather than the type of surgery, putting in mind that the type of analgesia given to surgical inpatients at our center was statically significant with ileus development in this study ( *p* =0.001).

This article primarily focuses on postoperative analgesics as a risk factor for ileus development among patients admitted to our center. However, the literature expands for other risk factors, such as the amount of blood loss. Artinyan and coworkers found a relationship between intraoperative blood loss and ileus. 25 Increased intraoperative blood loss can potentially lead to increased sympathetic and endocrine stress which in turn inhibit gastrointestinal transit similar to the mechanism by which pain enhances POI development. 25 Amount of bowel manipulation is yet another risk factor for ileus. Kalff et al found a correlation between bowel manipulation and ileus and concluded that the degree of gut paralysis to cholinergic stimulation is directly proportional to the degree of trauma, that is, bowel manipulation. 32 Looking at the greater picture, increased blood loss is in fact an indicator for bowel trauma and/or a heightened inﬂammatory response that may directly cause a prolonged POI. 25 All these factors and others, such as body mass index (BMI), comorbidities, and length of surgery, 25 26 32 are important parameters to look for in calculating the risk for POI, unfortunately these factors were out of the scope of this manuscript. Thus, a more detailed data collection involving the intraoperative notes and follow-up is recommended to discover the extent of these factors' involvement in POI development at our center or others in developing countries.

## Conclusion

A mix of analgesics (opioids and nonopioids) constituted most of the regime at our center. The majority of the surgical inpatients reported having moderate-to-severe pain on both scales used to measure pain in this study. Ileus incidence following abdominal surgeries (61%) was significantly higher than the reported incidence worldwide (10–30%). Our results found an association between analgesics that included opioids and a higher incidence of POI. Further studies should look at the doses of opioids used and whether alternative analgesic methods might result in less ileus. Patients who were receiving opioids were better able to ambulate postoperatively; however, this finding cannot be taken for granted due to the poor control on the confounding variables associated in this study.
